# Screening Tools for Mental Disorders Among Female Refugees: a Systematic Review

**DOI:** 10.1007/s40653-021-00375-9

**Published:** 2021-07-30

**Authors:** Orla Donnelly, Gerard Leavey

**Affiliations:** 1 Bamford Centre of Mental Health & Wellbeing, Ulster University, Coleraine, Northern Ireland; 2 Maastricht University, Maastricht, Netherlands

**Keywords:** Mental disorders, Screening tools, Female refugee

## Abstract

Female refugees are particularly vulnerable to mental disorders but assessment may be complex and challenging. Various screening tools have been developed for this population, but little is known about their usefulness. The main aim is to examine the literature on the effectiveness of screening tools for mental health problems among female refugees.

Systematic review of PubMed, PsycINFO and Embase to locate all published work. Comprehensive search terms were used and inclusion and exclusion criteria were formulated.

The initial search yielded 877 articles, of which 757 were removed after titles and abstracts were reviewed. Then, 121 full-text versions of articles were examined and 96 excluded according to the criteria. A total of 25 articles were included in this systematic review in accordance with the PRIMSA guidelines. Twenty screening tools were evaluated.

There is a lack of tools used to screen refugee women, and in particular those in emergency settings. Cultural factors may not be accounted for in the development of screening instruments**.** Further research in this field can help inform public health policies to address social, educational and occupational inclusion for refugee women in different contexts.

## Introduction

Worldwide, there are currently 25.9 million refugees with an additional 3.5 million asylum seekers (UNHCR, 2019). The displacement of people has significant effects worldwide and in particular health implications; physically, mentally and socially. The reported prevalence of mental disorders among refugees ranging from 20—80% (Song & Teichholtz, 2019). Women and girls constitute up to 48% of the refugee population and many be particularly vulnerable' (Lori & Boyle, 2015; Pavli, & Maltezou, 2017; Starck et al., 2020). In the context of war and persecution, women face many severe threats including rape, trafficking, domestic abuse and gender-specific trauma comprising of forced marriage, genital mutilation and coerced abortion (Starck et al., 2020). In addition, while women have limited access to sexual and reproductive health services throughout the refugee process, it is particularly evident in perinatal and antenatal care (WHO, 2019). Moreover, caregiving for the family tend to be borne by mothers (Lori & Boyle, 2015).

Assessment is fundamental in psychological practice to ensure that mental health problems are diagnosed and then addressed appropriately, quickly and accurately, identifying high risk individuals and subsequently improving treatment outcomes (Davidson et al., 2010). Screening tools are integral part of mental health assessment and help to avoid lengthy and costly clinical diagnostic assessment. A wide range of screening tools exist including questionnaires and structured interviews which help identify individuals who may be at risk (Peterson, 2019). However, considerable variation in culture, language and experience makes the development and application of mental health screening tools challenging (Zipfel et al., 2019). To date there has been limited assessment of mental health screening tools for the refugee women. Davidson et al. (2010) conducted a systematic review of mental health assessment for adults, adolescents and children and Gadeberg et al. (2017) undertook a review on validated screening and measurement tools for refugee children and youth. An overview on the mental health assessment of refugee women has yet to be completed. Thus, we aimed to identify and evaluate the current instruments for screening refugee women for mental health conditions.

## Exposure and Risk

Refugees from war-torn countries are exposed to traumatic events including imprisonment, torture, assault and loss of family members and property. Gender-based violence (GBV) has been linked to PTSD with victims reporting psychological issues including depression, sense of powerlessness and flashbacks (Lori & Boyle, 2015); Hameed et al., 2018). Between 100,000 to 250,000 women are estimated to have been sexually assaulted by men within a 3-month period during the Rwandan genocide (UN, 2014). Moreover, according to the UN, 70% of migrants travelling through north Africa to Europe have become victims to human trafficking with traffickers mainly targeting women and girls (UNODC, 2018). The living conditions within informal settlements and refugee camps often lack basic food and sanitation (Zaman et al., 2019). Refugee women may also experience racism and discrimination, and have difficulties integrating and settling in the host country (Freedman, 2016).

Rates of PTSD within refugee populations can range from 4–86% and depression from 5–31% (Bolton, 2019). Hameed et al. (2018) revealed females were more likely to have an established mental health diagnosis in comparison to males and were more likely to exhibit PTSD symptoms (Hameed et al., 2018). However, many mental health disorders are not explicitly or consistently assessed among refugee women (Killikelly et al., 2018). It is important to consider the maternal needs of refugee women and the likelihood of perinatal and postnatal mental health disorders. A study carried out in New England, USA revealed that refugee women had higher levels of postpartum depression (Tobin et al., 2015). Other evidence indicates that poor maternal mental health in pregnancy and the postpartum phase increases the likelihood that children faced suboptimal behavioural, cognitive and socio-emotional development (Kingston & Tough, 2014).

Crucially, in addition to the considerable variation in pre, and post-flight experiences, assessment requires an understanding of refugee heterogeneity relating to national, cultural, ethnic and religious backgrounds and their socioeconomic status and education levels (Rosenthal, 2018). These differences influence refugee symptom perception, health behaviors and help-seeking (Ghane et al., 2010). Explanatory models of mental illness project personal and social implications on the illness experience and are predominantly shaped by culture (Kleinman, 1978). Obtaining appropriate and timely assistance from ‘external’ and culturally insensitive agencies may be problematic and potentially harmful (Gadeberg et al., 2017). Due to specific considerations that apply to women specifically, an independent systematic review of screening tools is required. While mental health assessment and screening for refugees needs a specialized approach (Sharma et al., 2004). these are acknowledged to be beset with methodological problems (Bolton, 2019). Currently, there is no consensus on how to adapt tools for use in different cultural settings (Petkari, 2015).

Aims: (1) To identify and critically evaluate the effectiveness of screening tools used to detect mental health conditions within female refugee populations across different countries and cultures. (2) To assess cultural appropriateness of these screening tools. and (3) their utility in diverse contexts.

## Registration with Prospero

This review was registered on the PRSPERO database, CRD42020209689.

## Methods

A systematic review of all relevant published studies which included a primary electronic search on databases on PubMed, PsycInfo and Embase. We additionally explored the grey literature for unpublished studies. A broad searching strategy was applied using alternative terms and concepts which address the same question. This was especially true in regard to the broad symptomatology of refugees and their response to treatment as according to Gadeberg et al. (2017) they recommended to not focus solely on PTSD when assessing the mental health needs of refugees.

### Search Terms

The search terms used for this systematic review included *refugee* OR *asylum seeker* OR *displaced person* AND *women* OR *female* OR *woman* AND *mental health* OR psychiatric OR psychological OR *mental disorders/disease* OR *post-traumatic stress disorder/PTSD* OR *depression* OR *anxiety* OR *perinatal* OR *postnatal maternal health* OR *postpartum* OR *perinatal* OR *puerperal AND screening* OR *assessment* OR *instrument* OR *measurement* OR *questionnaire* OR *survey* OR *psychometric.* In addition, a secondary search was conducted through other scientific sources including Google Scholar and Maastricht University library and references of the retrieved articles were reviewed to reduce publication bias. From this, duplicates were identified and removed using Mendeley. The titles and abstracts of the remaining articles were reviewed and all non-relevant articles excluded according to the exclusion and inclusion criteria in accordance with the PRISMA guidelines Then, the full-text version of articles was examined and excluded according to the criteria. A flow chart of study identification based on the PRIMSA guidelines will be created.

Inclusion and exclusion criteria was formulated based on the research question and is explicitly stated. This study focuses on women of refugee background in high, middle and low income settings. An article was included in this research if it; (a) clearly states the effectiveness of mental health screening tools used (b) sample size with a proportion of 50% or more women in the study population. Gagnon et al. (2004) reasoned that women should constitute at least 50% of the sample when dealing primarily with women’s health measurements (Gagnon et al., 2004). (c) population of refugee or asylum seekers (d) in the English language (e) time frame of 2000 to 2020. Articles which were excluded from this systematic review were: (a) duplicate reports (b) articles which studied screening tools in predominantly male sample populations or among children and adolescents (c) population is not of refugee background (d) lack of focus on mental health screening instruments (e) articles not in English (f) articles which were not available. The cases which were “borderline” were carefully considered with discussion and shared decision making with a supervisor.

The studies which are included in this systematic review was assessed by one reviewer through a quality assessment checklist provided by The National Institute for Health and Care Excellence (NICE) (NICE, 2012). This checklist evaluated the theoretical approach, study design, data collection methods, validity, analysis and ethical approvals of the included articles. The overall quality assessment was ranked through high, medium and low quality classifications. A second reviewer checked and validated the chosen studies. Disagreements regarding quality assessment were resolved by discussion with a second reviewer.

Data were extracted from the studies based on study characteristics including study date, study author, study title, study country, study design, description of the population, sample size, gender distribution and the screening tool used. The level of cultural adaptation is assessed using a conceptual model developed by Okamoto et al. (2014). Four levels of adaptation were coded using this criterion from (1) no adaptation to (4) culturally grounded adaptation. Each article was coded according to the following criteria; (1) No adaption of screening tool but direct translation was used using standardised translation techniques with consensus from experts or locals, (2) Surface adaption: minor changes were made to the content of the original screening instrument for the purpose of incorporating cultural expressions or belief, (3) Deep structural adaptation: use of systematic methods to develop culturally appropriate questions and content in addition to the original questionnaire. For example, additional items of new content or questions with methods including focus groups and interviews, (4) Culturally grounded adaptation: the development and refinement of a new, unique screening instrument specifically tailored to a certain cultural group (Killikelly et al., 2018). Disagreements regarding data extraction were resolved by discussion with a second reviewer. Data analysis is displayed using tables and figures to evaluate the studies included.

## Results

**Fig. 1:**
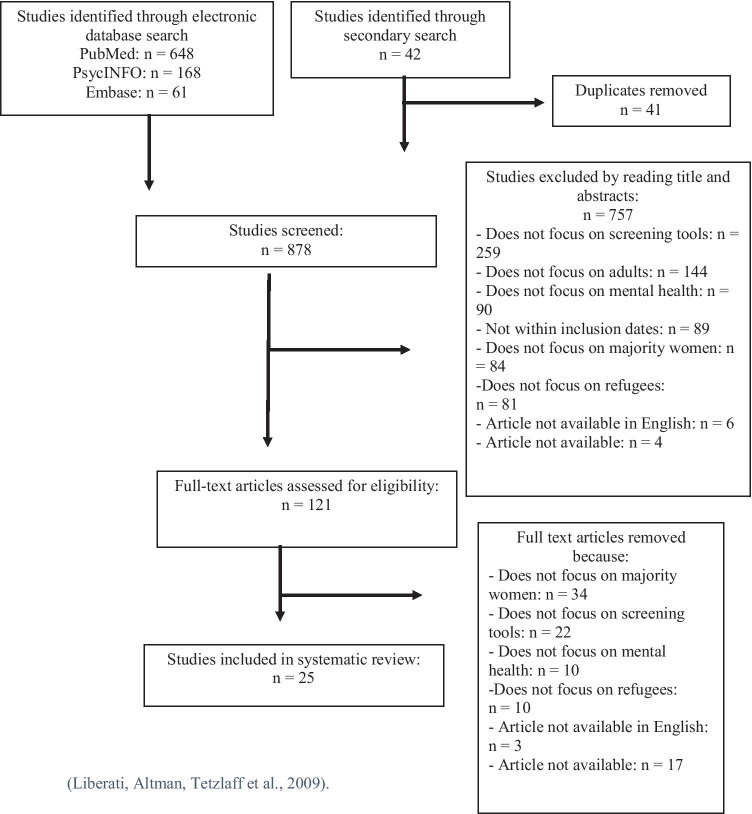
Flow chart of study identification based on the PRIMSA guidelines

Through the primary electronic search, a total of 877 were found which included PubMed (n = 648), PsycInfo (n = 168), and Embase (n = 61) and 42 additional articles were found through the secondary search. Figure 1 shows a flow chart of study identification which is based on the PRISMA guidelines. We identified and removed 41 duplicates. The remaining 878 studies were screened and 757 were removed after titles and abstracts of the remaining articles were reviewed and all non-relevant articles excluded according to the criteria. Then, 121 full-text versions of articles were examined and 96 excluded according to the criteria. A total of 25 articles were included in this systematic review.

The included studies reported on the effectiveness of these screening tools. The accessibility, acceptability, cultural appropriateness, administration and barriers of screening instruments were integrated within the articles which occurred in a wide range of settings among different refugee populations. In total, twenty different mental health screening tools were noted to have incorporated adaptations of validated screening tools (n = 16) and newly developed screening tools (n = 4). The studies were published from 2003 – 2020 and the sample size ranged from 4 to 810 participants consisting of women over the age of 14 years old. The studies selected included both qualitative (n = 9), quantitative (n = 10) and mixed method (n = 6) research designs.

### Study Population

Seven studies focused on females only, and eighteen studies had a majority female population (> 50% of female participants).; age ranged from 14 to 89 years old. Places of origin were:—Europe (Bosnian, Russian), Middle East (Iraqi, Yazidi, Palestinian), Asia (Burmese, Bhutanese, Karen) and Africa (Somalian, Congolese).

### Settings and Administration

In all the articles, the refugee women were assessed in the receiving country, predominantly represented by the United States (n = 14). Other studies were also carried out in different high income countries including Canada (n = 2), Australia (n = 1), England (n = 1) and Germany (n = 1). One study was carried out in South Africa and the setting of three studies was refugee camps in low income countries including Rwanda (n = 2), Ethiopia (n = 2) and Lebanon (n = 1). In the included studies, women were assessed in a range of different environments. These encompassed home visits, primary health care settings such as outpatient medical clinics and general practice, community centers, places of recreation and religious places of worship as well as humanitarian settings in refugee camps in low resource countries. A wide range of health professionals performed the mental health screening including psychiatrists, psychologists, nurses, midwives, community health workers, refugee counsellors and social workers. Interpreters were also present in five screening programs.

### Characteristics of Screening Tools

A total of twenty screening tools were used to measure the mental health of refugee women in different settings with three studies including more than one screening tool. The screening tools used in these articles can be seen in the Table 1 below.

**Table 1:** Characteristics of the included screening tools that have been used in refugee women populations

**Study**	**Screening Tool Used**	**Measurement approach**	**Language**	**Study Population and Sample Size (total number / % women)**	**Design**
Ovitt et al. (2003)	HSCL-25	Depression and anxiety	Bosnian	Bosnian refugees in the US 8 / 50%	Qualitative—Client questionnaire and structured discussion
Hoffmann et al. (2005)	SF-12	Various mental health disorders including depression, PTSD, adjustment disorder, bipolar disorder and psychosis	Russian	Russian Refugees in the US 52 / 63%	Qualitative—Unstructured Interviews and questionnaire
Bhui et al. (2006)	MINI International Neuropsychiatric Interview	CMDs	Somalian	Somalian refugees in England 143 / 50.3%	Mixed Methods
Shoeb et al. (2007)	HTQ	PTSD symptoms	Arabic	Iraqi refugees in the US 60 / 50%	Qualitative—Ethnographic interviews
Hollifield et al. (2012)	RHS-15	Anxiety, depression and PTSD symptoms	Burmese, Bhutanese And Iraqi	Burmese, Bhutanese And Iraqi Refugees in the US 251 / 50%	Quantitative—Cross sectional design
Hinton et al. (2013)	CSSI	PTSD symptoms	Khmer	Cambodian refugees in the US 226 / 66%	Qualitative—Survey and Questionnaire
Johnson-Agbakwu et al (2014)	RHS-15	Emotional Distress	Arabic, Burmese, English, Karen, Nepali, Somalian	Multi-ethnic sample of refugees in the US 112 / 100%	Mixed Methods
Bell et al. (2015a, 2015b)	SRQ-20 SRQ-SIB	CMDs	Kinyarwanda	Congolese refugees in Rwanda 810 / 100%	Qualitative -Questionnaires
Tobin et al. (2015)	PDPI-R	Postpartum Depression	English	Refugees in the US 126 / 100%	Quantitative—Retrospective chart-review
Bell et al. (2015a, 2015b)	Self-Report Questionnaire-5	CMDs and suicide ideation	Kinyarwanda	Congolese refugees in Rwanda 810 / 100%	Quantitative—Cross-sectional design
Ferrari et al. (2016)	iCCAS	CMDs	English, Spanish	Refugees in Canada 74 / 66%	Mixed Methods -RCT and Survey
Biegler et al. (2016)	HSCL-25 HTQ	Depression and PTSD symptoms	Khmer	Cambodian refugees in the US 331 / 64.4%	Quantitative—RCT
Brink et al. (2016)	Karen Mental Health Screener	Symptoms of depression and PTSD	Karen	Karen refugees in the US 180 / 70%	Qualitative—Interviews
Polcher & Calloway (2016)	RHS-15	Emotional distress	Iraqi, Nepali, Bhutanese, Karen, Burmese	Multi-ethnic refugees in the US 179 / 59%	Qualitative -Questionnaire
Tomita et al. (2016)	QIDS	Symptoms of Depression	English	Multi-ethnic Refugees in South Africa 135 / 50.3%	Quantitative—Longitudinal cohort design
Gerdau et al. (2017)	MINI International Neuropsychiatric Interview	PTSD and related disorders	German	Yazidi refugees in Germany 4 / 100%	Mixed Methods
Lepper et al. (2017)	Primary Care Behavioural Health Screener	Behavioural health problems	Bosnian	Bosnian refugees in the US 131 / 57%	Quantitative—Prospective cross-sectional design
Llosa et al. (2017)	WASSS-H WASSS-I VOLTAC SRQ	Severe distress or mental disorders	Arabic	Palestinian refugees in Lebanon refugee camp 283 / 50.9%	Mixed Methods
Salt et al. (2017)	RHS – 15	CMDs	Somalian, Nepalian, English, Karenni	Multi-ethnic refugees in the US 12 / 100%	Mixed Methods
Getnet & Alem (2018)	CES-D	Depression	Tigrigna	Eritrean refugees in Mai-Aini refugee camp in Ethiopia 562 / 54.1%	Quantitative—Cross-sectional survey
Ferrari et al. (2019)	iCCAS	CMDs	English, Spanish	Multi-ethnic Refugees in Canada 10 / 60%	Qualitative Semi-structured Interviews
Sorkin et al. (2019)	HSCL-25 HTQ	Major Depressive Disorder (MDD) and PTSD symptoms	English, Khmer	Cambodian refugees in the US 331 / 66.7%	Quantitative—RCT
Willey et al. (2019)	EPDS	Perinatal depression	English, Dara, Burmese, Vietnamese	Asian refugees in Australia 22 / 100%	Qualitative—Focus group discussion and semi-structured interviews
Getnet & Alem (2019)	SoC-13 scale	Measure of resilience	Tigrigna	Eritrean Refugees in Ethiopia 562 / 54.1%	Quantitative—Cross-sectional survey
Baird et al. (2020)	RHS-15	CMDs	English, Burmese, Arabic, Swahili, Nepali, Somali, Farsi, Karen, Kinyarwanda, Mexican Spanish	Refugees in the US 352 / 53.9%	Quantitative—Retrospective analysis

Hopkins Symptoms Checklist 25 (HSCL-25)

12-Short Form health survey questionnaire (SF-12)

Harvard Trauma Questionnaire (HTQ)

Refugee Health Screener-15 (RHS-15)

Cambodian Somatic Symptom and Syndrome Inventory (CSSI)

Self-Reporting Questionnaire (SRQ- 20)

Self-Reporting Questionnaire-Suicidal ideation and Behaviour (SRQ-SIB)

Postpartum Depression Predictor Inventory Revised (PDPI-R)

Interactive Computer-Assisted Client Assessment Survey (iCCAS)

Quick Inventory of Depressive Symptomatology (QIDS),

WHO-UNHCR Assessment Schedule of Serious Symptoms in Humanitarian Settings-Household Interviews (WASSS-H)

WHO-UNHCR Assessment Schedule of Serious Symptoms in Humanitarian Settings- Individual Interview (WASSS- I)

Vignettes of Local Terms and Concepts (VOLTAC)

Center for Epidemiological Studies Depression Scale (CES-D)

Edinburgh Postnatal Depression Scale (EPDS)

Sense of Coherence scale (SoC-13)

Most screening tools were administered using questionnaires or interviews. Five studies were interactive based screening tools which were carried out using computers, iPads and mobile phones. The screening tool measurement approach varied. Twenty-two articles screened for Common Mental Disorders (CMDs) which incorporates PTSD, generalized anxiety disorders, panic disorders, phobias, social anxiety disorder and obsessive–compulsive disorder (OCD) (National Collaborating Centre for Mental Health, 2011). Only two studies covered maternal mental health conditions, postpartum depression (n = 1) and perinatal depression (n = 1). One study focused on resilience measurement. The number of items in the screening tools varied between 5 and 52 items.

### Language and adaptations

**Fig. 2:**
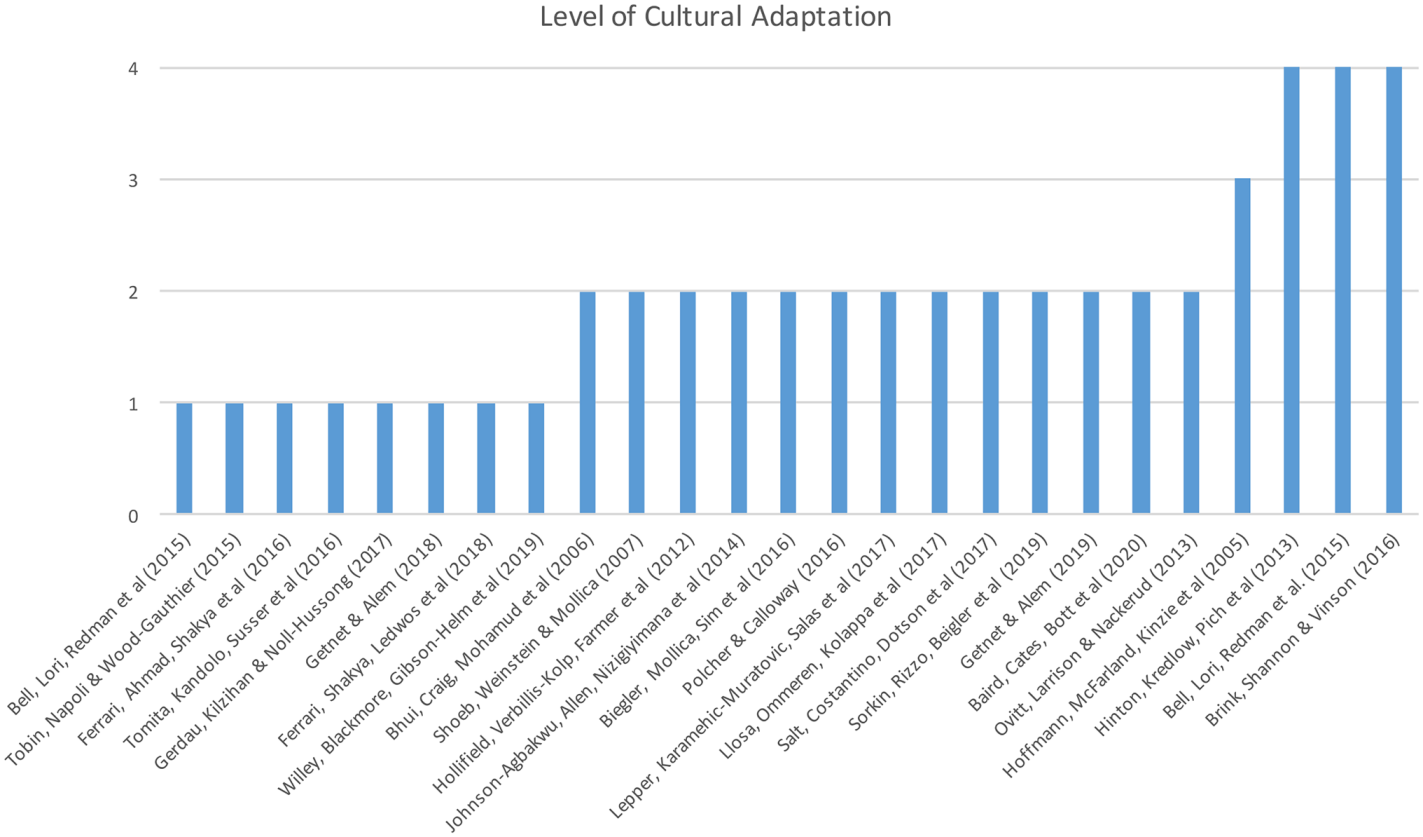
Level of cultural adaptation of included studies according to Okamato et al. (2014)

Most tools used were adapted to suit the served populations; 18 of these translated into the native language of the refugees, four were presented in a bilingual form and three were in English (Table 1). Most tools were translated using a combination of cross-cultural methods including back-translation, bilingual technique, pre-testing and committee evaluations with expert translators. Of the twenty-five articles, interpreters were noted in five of the screening programs. Using the model of cultural adaptation by Okamoto et al. (2014), eight screening instruments had no cultural adaptation, thirteen had surface adaptations, one had deep structural adaptation and three were culturally grounded (Fig. 2). Certain screening tools have already been validated in other studies and contexts including the HSCL, HTQ, RHS-15, WASSS, VOLTAC, PDPI-R and SRQ. Other screening tools; MINI International Neuropsychiatric Interview, SRQ-SIB, Karen Mental Health Screener were validated against other screening tools including the Structured Clinical Interview for DSM-IV Axis I Disorders (SCID), New Mexico Refugee Symptom Checklist-121 and the RHQ.

## Discussion

We sought to review the literature on mental health screening tools used for women of refugee background in different settings with a focus on their acceptability and accessibility. Although we found considerable variation in the different methods used to evaluate the screening tools, with one exception (Tobin et al., 2015), all appeared to be useful. Nevertheless, we noted a range of weaknesses in the approaches to screening for mental health problems in this population, including limitations to translation and cultural adaptation of tools.

The inclusion of culturally inappropriate symptoms in screening measurements may lead to misdiagnosis and delayed care, unsuitable treatment and unnecessary referrals (Brink et al., 2016). Thus, what is commonly understood in Western settings may be incomprehensible elsewhere. The DSM-IV described culture-bound syndromes as indigenously regarded ‘illnesses’, comprising localised diagnostic categories that provide a framework to comprehend certain repetitive, patterned, and troubling sets of experiences and observations. For example, *Latah* in Malaysia, or *ataque de nervios* in Latin American societies are commonly cited examples of such syndromes. Conversely, eating disorders, increasingly prevalent in developed capitalist societies are unlikely to be recognisable in LMICs. The terminology for some disorders may also be ambiguous and confusing. For example, the Somali term for severe depression also means severe headaches and migraine (Bhui et al., 2006). We noted that studies with a higher level of cultural adaptation (Fig. 2) used a combination of translation techniques. For example, the CSSI tools consisted of a symptom checklist with more culturally suitable idioms of distress to portray symptoms of mental illness among Cambodian refugees.

The issue of the applicability and appropriateness of Western measures for assessing clients from non-Western backgrounds appeared throughout the reviewed articles. PTSD has been criticized as lacking validity and regarded as a Western construct (Gadeberg et al., 2017), (Summerfield, 2008), inapplicable in non-Western settings and populations. Thus, while traumatic events may have psychological consequences, the symptoms may be experienced and expressed differently across different cultures (Alford, 2016) and may require alternative assessments and interventions (Tobin et al., 2015). There are concerns about the implicit reductionism of PTSD category whereby it has the potential to medicalise human suffering and overshadow the social and moral implications of war or genocide. Setting aside any moral implications, the content validity of a PTSD diagnosis is questioned due to the absence of somatic symptoms, commonly featuring among non-Western cultural groups.

Qualitative methods may provide insights into culture-specific beliefs, experiences and presentation of illness, informing and/supplementing the use and development of screening tools. The use of Likert type scales to examine the complex and highly nuanced cultural belief systems are likely to be reductionist and thus inappropriate (Baird et al., 2020) and thus, Hinton et al. (2013) recommended the use of somatic symptom inventories to use alongside psychometric instruments.

While the newly developed screening tools were validated against other screening instruments, further validation of these tools is warranted. While ‘gold standard’ instruments exist for use in Western populations, these doesn’t necessarily transfer across different languages and cultures and comparison between results are complicated (Gadeberg et al., 2017). Criterion validity may be the most dimension of validity for cross-cultural work, referring to the validity of an instrument judged by comparing its performance with that of a "gold standard" that is, a robust and irrefutable standard of evidence that a certain disease exists.

For example, while the HTQ is an acknowledged and widely used assessment tool in trauma exposure and trauma-related symptoms in refugees (Berthold et al., 2018) its usefulness across all refugee contexts has been questioned (Shoeb et al, 2007; Getnet & Alem, 2019; Salt et al., 2017).

## Emergency Settings

Health services for refugee families within temporary camps are often organised by non-government organisations and delivered by volunteers with variable levels of training (Hermans et al., 2017; Llosa et al., 2017; Shoeb et al., 2007). The evidence suggests that the WASSS can assist detection of those with the greatest mental health needs (Llosa et al., 2017). The SoC-13 which was tested in an Ethiopian refugee camp has been used by psychiatrists, counselors and social workers in non-clinical settings (Getnet & Alem, 2019). The SRQ-5 has also proved useful in low resource settings as it clinically identified common mental health disorders and suicide ideation in a female refugee population. While further evaluation of this tool is necessary, it has been used in a women’s health clinic and primary care settings in refugee camps (Bell et al., 2015a, b).

Another promising approach was that used by Llosa and colleagues (2017) adopting a two phase, screen-confirm method to identify individuals with severe mental health disorders. This permitted the application of standardized measures in a context where limited time and resources blocked the development and validation of culturally appropriate screeners (Llosa et al., 2017)

## Use of E-Mental Health Screening Tools

Interactive eHealth screening tools may overcome the communication and stigma issues associated with mental health screening (Fonseca et al., 2016). Currently, this technological approach has been implemented in certain contexts to increase access and utilization of screening programs for refugee women. Ferrari et al. (2016) conducted a tablet-based touch screen survey among refugees in a Canadian primary care setting which demonstrated agreement and positive attitudes towards the interactive self-assessment tool, iCCAS. The use of the iCCAS technological screener increased client ease and comfort in reporting mental distress in comparison to face-to- face interviews as clients were more willing to discuss their concerns through self- assessment (Ferrari et al., 2016). In addition, a study carried out in South Africa used short message service via mobile phones to screen for the risk of depression in refugee populations which appeared to be feasible and acceptable as clients preferred the anonymity of this service, reducing stigma associated with help-seeking behaviours. (Tomita et al., 2016). The use of technology may also increase the accessibility to different languages, helping to overcome the linguistic barriers of the population served in community health care practices (Ferrari et al., 2016). In addition, one challenge identified in the administration of screening tools was the lack of professionals available to administer the screening tools. Ovitt et al. (2003) discussed the need for professionals to analyse instruments in a timely fashion as well as the lack of bilingual mental health workers who could provide this service. In many contexts, this is lacking and therefore affected the administration and implementation of mental health screening. Innovations to accommodate refugees should be explored including application-based screening in different languages or the use of audio or picture options to transcend the linguistic and communication barriers which exist in clinical service settings.

## Maternal mental Health

Overall, we noted a lack of screening tools which targeted maternal mental health assessment with only two of the articles focusing on maternal mental health. In a study carried out among Afghan mothers in two Pakistani refugee camps, 36% of mothers screened positive for CMDs with 91% of these women having suicidal thoughts over the course of a month (Rahman & Hafeez, 2003). This highlights the prevalence and severity of mental distress among refugee women caring for their young families in refugee camps. Tobin et al. (2015) reported that the use of the PDPI-R screener among a multi-ethnic group of refugees in New England did not prove to be effective as the estimated levels of postpartum depression was much lower than expected despite the presence of significant stressors related to postpartum depression. Nurses were seen as a crucial link in the provision of maternity services as they have the ability to identify women at risk of postpartum depression in the early stage. However, the quality of the relationship is central to effective screening as the mother needs to feel content to effectively complete the questionnaire (Tobin et al., 2015). Additionally, the role of interpreters was highlighted by Willey et al. (2019) in a study among Asian refugees in Australia in which a small group of female interpreters acted as “cultural brokers” facilitating discussion between the mothers and midwives (Willey et al., 2019). There were no studies which were carried out in low, and middle income countries, revealing a major gap in mental health care in female populations.

Maternal health care needs to be effectively implemented into routine maternity care as early identification and management of mental health conditions is crucial to improve not only maternal outcomes but also neonatal outcomes.

## Conclusion

As far as we can tell, this is the first review to assess the use of mental health screening tools for women refugees. Importantly, while we noted an increasing use of such tools across a wide range of refugee settings and contexts, more work is required on their validation, training for use and barriers to implementation. This is particularly true for use in the area of perinatal mental health which appears to be greatly underserved. The importance of understanding and incorporating the cultural beliefs and idioms of distress cannot be understated. Moreover, all cross-cultural research requires robust translation and transparent validation methods.

Lastly, although there are obvious barriers to using digital health technology in conflict and other challenging environments, their potential may be paradigm-chnaging, as technology becomes cheaper and more available, helping to bring rapid and professional assessment and consultation through remote access. This will be a major benefit to women, their children and communities.

### Limitations

The articles were limited to those written in English and were published from the year 2000 to present. In some cases, we were unable to extract full data from all included studies as some do not disclose all data collection methods and study populations were vague. A comparison of screening tools was difficult due to their heterogeneity. Nevertheless, some key strengths of this study was the diversity of the refugee assessments analyzed, representing numerous different language and cultural versions. For this systematic review, a broad search strategy was used, and search terms were based on recommendations by researchers within the mental health field to locate all relevant published and unpublished work.
